# Polydopamine-Modified Boron Nitride Reinforced Silicone Gel Composites with Enhanced Thermal Conductivity and Electrical Insulation Performance

**DOI:** 10.3390/gels12070644

**Published:** 2026-07-19

**Authors:** Mengjia Feng, Chaoyue Zhao, Wenbo Li, Xinfeng Lv, Zichen Cui, Jianzeng Guo, Mai Hao

**Affiliations:** 1 State Key Laboratory of Smart Power Distribution Equipment and System, Hebei University of Technology, Tianjin 300123, China; 202431402163@stu.hebut.edu.cn (C.Z.); 202531402049@stu.hebut.edu.cn (W.L.); 202531402010@stu.hebut.edu.cn (Z.C.); 202511401012@stu.hebut.edu.cn (J.G.); 202521401045@stu.hebut.edu.cn (M.H.); 2 School of Electrical Engineering, Hebei University of Technology, Tianjin 300123, China; 3 School of Mechanical Engineering, Hebei University of Technology, Tianjin 300123, China; 222452@stu.hebut.edu.cn

**Keywords:** silicone gel, boron nitride, thermal conductivity, electrical insulation, partial discharge

## Abstract

Silicone gel (SG) is widely used as a soft encapsulation material for high-voltage power devices because of its excellent flexibility, thermal stability, and electrical insulation. However, its intrinsically low thermal conductivity and susceptibility to partial discharge (PD) at triple-junction interfaces restrict long-term operational reliability. In this study, polydopamine-modified hexagonal boron nitride (P-BN) was introduced into silicone gel to construct thermally conductive and electrically insulating composites. The SG/P-BN composites exhibited reduced filler agglomeration and a more continuous filler–matrix morphology than the corresponding SG/BN composites, while the model-extrapolated trap analysis suggested composition-dependent changes in the higher energy charge trapping states of the P-BN-containing composites. As a result, the SG/P-BN composites exhibited enhanced thermal stability, reduced coefficient of thermal expansion, and improved heat-transfer capability, with thermal conductivity increasing from 0.183 W/m·K for pristine SG to 0.25 W/m·K. The composite containing 2 wt% P-BN showed the best insulation performance, with breakdown strength increasing from 24.05 to 28.45 kV/mm at 25 °C and from 19.59 to 24.71 kV/mm at 150 °C. Under a simplified triple-junction laboratory configuration, the PD inception voltage increased from approximately 3.1 kV for pristine SG to 4.1 kV for SG/P-BN_2_, accompanied by fewer high-amplitude discharges. This work demonstrates improved material-level thermal conductivity and electrical insulation performance of P-BN-containing silicone gel composites under the investigated laboratory conditions.

## 1. Introduction

With the rapid development of wide-bandgap semiconductor devices represented by silicon carbide (SiC) and gallium nitride (GaN), power modules have been increasingly applied in high-power fields such as flexible direct-current transmission, new energy vehicles, rail transit, and aerospace, while continuously evolving toward higher voltages, higher power densities, and elevated operating temperatures [1,2,3,4,5]. These trends impose unprecedented requirements on the electrical insulation, thermal conductivity, and service reliability of encapsulation insulating materials. Silicone gel (SG) has been widely used as a soft encapsulation insulating material owing to its excellent flexibility, electrical insulation properties, and good processing compatibility [6,7,8,9]. However, in practical power module packaging structures, triple-junction interfaces are inevitably formed among ceramic substrates, metal electrodes, and encapsulation media. Due to dielectric constant mismatch, geometric edge effects, and interfacial microdefects, these regions are prone to local electric-field enhancement, which can induce partial discharge, electrical treeing, and interfacial breakdown, thus becoming critical weak points for insulation failure [10,11,12,13]. Meanwhile, the intrinsically low thermal conductivity of pristine SG limits efficient heat dissipation during high-power operation. Under coupled thermal and electrical stresses, enhanced carrier transport and space charge accumulation can further accelerate insulation degradation, especially at triple-junction interfaces [14,15,16,17]. Therefore, the development of SG-based composite encapsulation materials with simultaneously improved thermal conductivity, regulated carrier transport, and enhanced partial-discharge resistance is of great significance for improving the operational reliability of next-generation high-voltage power modules.

Current strategies for improving the performance of silicone gel encapsulation materials mainly involve molecular structure modification and filler compounding. Molecular structure modification is generally achieved by introducing functional groups, such as phenyl groups, into silicone side chains through chemical synthesis, thereby enhancing chain rigidity, thermal stability, and electrical insulation performance [18,19]. However, this approach usually requires complicated synthesis and purification procedures, strict reaction conditions, and careful control of possible byproducts, which may limit its scalability and compatibility with practical encapsulation processes [20]. By comparison, filler compounding provides a more straightforward and versatile route for tailoring the properties of silicone gel. Therefore, filler compounding has become one of the most widely adopted strategies for developing high-performance silicone gel encapsulation materials with improved thermal management and insulation reliability.

Inorganic and functional fillers have been widely employed to improve the thermal and electrical insulation properties of silicone-based encapsulation materials by constructing heat-conduction pathways, regulating electric field distribution, and introducing charge-trapping sites [21,22,23,24,25,26,27,28]. For instance, highly insulating fillers, such as SiO_2_ and nanodiamond, can provide charge-trapping centers and enhance breakdown strength [21]; ceramic fillers, such as AlN, are effective in improving thermal transport and resistance to thermal aging [22]; SiC fillers can impart nonlinear conductivity to composite systems, thereby reshaping the internal electric field distribution of power modules and increasing the partial discharge inception voltage [23]; and fluorinated graphene frameworks can form continuous thermal networks, leading to a remarkable enhancement in thermal conductivity [24]. Nevertheless, existing filler-reinforced systems still face challenges in achieving the synergistic optimization of thermal management and insulation reliability. Filler agglomeration and discontinuous inorganic/organic interfaces can induce local electric field concentration, thus weakening the insulation reliability of the composites [29,30]. Moreover, most previous studies have focused on intrinsic material parameters, using standard specimens and conventional electrode configurations. Limited attention has been paid to partial discharge behavior under device-relevant interfacial conditions, including triple-junction regions, metal edges, and ceramic substrate/encapsulant interfaces in power modules. In fact, an improvement in macroscopic breakdown strength does not necessarily guarantee enhanced partial discharge resistance in practical device structures [31].

Although the fillers discussed above can improve specific properties of silicone-based composites, their functional emphasis differs. SiO_2_ is mainly effective in interfacial and charge-trapping regulation, SiC is attractive for nonlinear conduction and electric-field grading, whereas highly conductive graphene-based networks can markedly enhance heat transfer but may be unfavorable for maintaining high electrical insulation. In comparison, hexagonal boron nitride (h-BN) combines high thermal conductivity, a wide bandgap, low dielectric loss, excellent electrical insulation, and good thermal stability, making it particularly attractive for SG composites requiring simultaneous thermal and electrical performance [32,33,34,35]. However, pristine h-BN is characterized by a lack of active surface groups and high interfacial energy, which leads to weak interactions between the filler and matrix while promoting strong filler–filler attraction. As a result, BN is prone to agglomeration in the silicone gel matrix and may form interfacial voids or dielectric discontinuities. These defects not only interrupt the continuity of heat-conduction pathways but also act as weak regions for local electric field enhancement, partial discharge development, and electrical breakdown. Therefore, improving the interfacial compatibility between BN and silicone gel is critical for simultaneously improving the thermal conductivity and electrical insulation properties of SG composites.

In this work, polydopamine-modified h-BN (P-BN) was incorporated into silicone gel to prepare SG/P-BN composite encapsulants. The structure, morphology, thermal properties, dielectric properties, volume resistivity, and temperature-dependent breakdown behavior of the composites were systematically investigated. In addition, a simplified triple-junction laboratory configuration composed of a sphere-plate electrode and a ceramic sheet was constructed based on the electrode/ceramic/silicone gel interface found in power-module packaging. The partial discharge inception voltage and phase-resolved discharge behavior of SG, SG/BN, and SG/P-BN were comparatively evaluated under this configuration. This study investigates the material-level thermal and electrical properties of P-BN-containing silicone gel composites together with their comparative partial-discharge behavior under the specified laboratory geometry.

## 2. Results and Discussion

### 2.1. Morphology and Characterization

**Figure 1:**
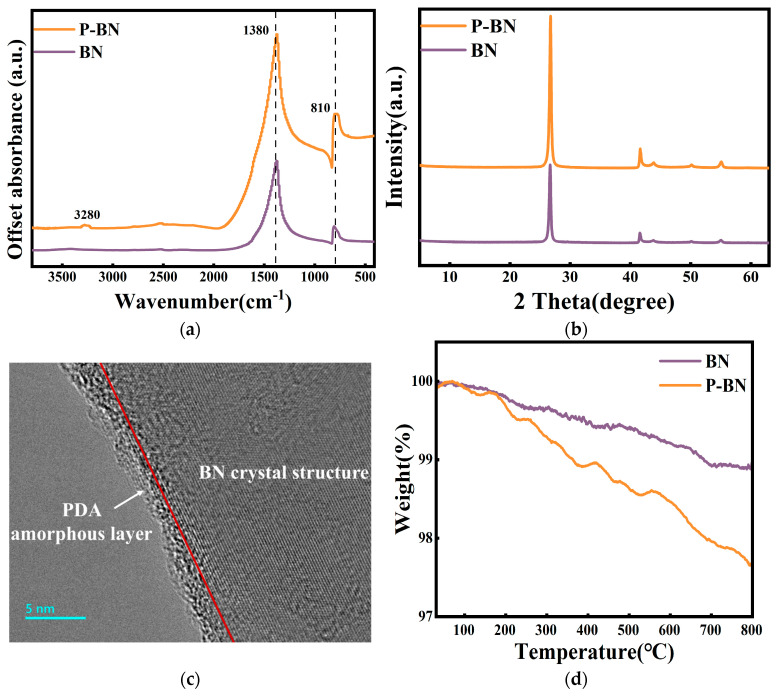
Structural and morphological characterization of pristine BN and P-BN. (**a**) FTIR spectra; (**b**) XRD patterns; (**c**) TEM image of P-BN showing the crystalline BN region and amorphous PDA coating layer; and (**d**) TGA curves.

To evaluate the surface changes in h-BN after dopamine-assisted treatment, crystalline structure, morphology, and thermal degradation behavior of BN and P-BN were characterized by FTIR, XRD, TEM, and TGA, respectively. Figure 1 summarizes the structural and morphological features of pristine BN and P-BN. In the FTIR spectra (Figure 1a), both BN and P-BN exhibit two characteristic absorption bands at approximately 1380 and 810 cm^−1^, corresponding to the in-plane stretching vibration of B-N bonds and the out-of-plane bending vibration of B-N-B bonds in h-BN, respectively. These bands indicate that the main framework of h-BN is retained after PDA modification. Compared with pristine BN, P-BN displays a weak and broad absorption band around 3280 cm^−1^, which is assigned to the O-H/N-H stretching vibrations of phenolic hydroxyl and amino groups in PDA. In addition, slight changes are observed in the regions of 1500–1600 cm^−1^ and 1200–1300 cm^−1^ which are associated with aromatic skeleton vibrations and C-N/C-O stretching vibrations from PDA. These spectral changes are consistent with the presence of a dopamine-derived organic coating on the BN surface without destroying the intrinsic chemical structure of h-BN.

XRD analysis was further performed to test the influence of PDA modification on the crystalline structure of h-BN. As presented in Figure 1b, both BN and P-BN exhibit a prominent diffraction peak at 2θ = 26.7°, corresponding to the (002) plane of h-BN, along with several weaker characteristic peaks located at 41–44°, 50°, and 55° [36]. After PDA modification, the main diffraction peaks of P-BN remain nearly identical to those of pristine BN, with no obvious peak shift or newly formed crystalline phase. This result indicates that the PDA coating process does not damage the hexagonal crystal structure of BN. In addition, no distinct diffraction peak associated with PDA is detected, which can be attributed to the amorphous nature and relatively low content of the PDA layer. Therefore, the XRD results confirm that P-BN retains the intrinsic crystalline structure of h-BN after surface modification.

TEM observation further provides direct morphological evidence for the formation of the PDA coating. In Figure 1c, clear and ordered lattice fringes are observed in the inner region of P-BN, indicating that the crystalline structure of BN is well maintained after surface modification. In contrast, a thin amorphous layer is visible along the edge of the BN nanosheet and is clearly distinguishable from the crystalline interior. This nanoscale layer is relatively uniform on the BN surface and can be assigned to the deposited PDA coating. These observations confirm that PDA is successfully coated onto the BN surface without disrupting the intrinsic crystalline structure of h-BN.

TGA was conducted to further verify the presence of the PDA layer on BN. As presented in Figure 1d, pristine BN exhibits negligible mass loss over the tested temperature range, indicating its excellent thermal stability. In comparison, P-BN shows more pronounced and continuous weight loss, particularly in the medium- and high-temperature regions. Since h-BN remains thermally stable under these conditions, the additional mass loss of P-BN is mainly attributed to the thermal decomposition of the organic PDA coating. This difference in thermal degradation behavior further supports the successful deposition of PDA on the BN surface.

Taken together, the FTIR, TEM, and TGA results provide complementary evidence for the presence of a PDA-associated organic surface layer on BN surface, while XRD analysis demonstrates that the intrinsic crystalline structure of h-BN is well preserved during the modification process. The PDA layer introduces hydroxyl, amino, and aromatic groups onto the BN surface while retaining the inherent structural and thermal stability advantages of h-BN. Such a surface-engineered structure may influence the dispersion and interfacial morphology of BN within the silicone gel matrix, thereby providing a possible basis for the observed thermal and electrical properties of the SG/P-BN composites.

**Figure 2:**
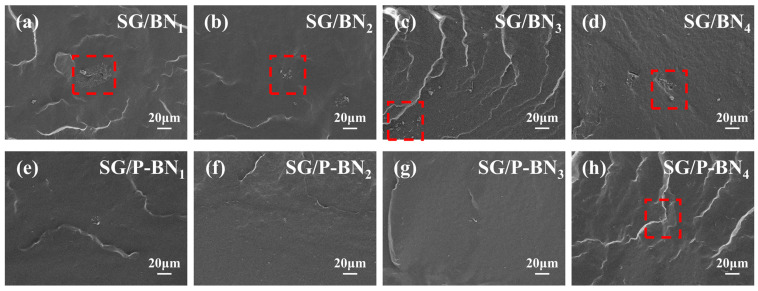
Cross-sectional SEM images of silicone gel composites filled with pristine BN and PDA-modified BN. (**a**–**d**) SEM images of SG/BN composites with different BN contents. (**e**–**h**) SEM images of SG/P-BN composites with different P-BN contents.

Following the confirmation of PDA modification on BN, SEM was further employed to examine the fractured cross-sectional morphology of the silicone gel composites, with emphasis on filler dispersion and filler–matrix interfacial compatibility. Figure 2 presents the fractured cross-sections of the SG/BN_1_-SG/BN_4_ and SG/P-BN_1_-SG/P-BN_4_ composites. For the composites filled with pristine BN, obvious flake-like agglomerates are observed within the silicone gel matrix. Some BN particles are locally clustered, accompanied by visible interfacial discontinuities between the fillers and the surrounding silicone gel. This agglomeration becomes more pronounced with increasing BN loading, suggesting that pristine BN is difficult to disperse uniformly in silicone gel because of its chemically inert surface and weak interaction with silicone molecular chains. Such filler aggregation can weaken filler–matrix interfacial bonding and generate microdefects or interfacial voids, which may interrupt heat-conduction pathways and serve as electrical weak regions under high electric fields.

In comparison, the SG/P-BN_1_-SG/P-BN_4_ composites show denser and smoother fractured surfaces, indicating that the dispersion of P-BN in the silicone gel matrix is significantly improved. Large filler agglomerates are markedly reduced even at relatively high filler loadings, and the filler–matrix interface appears more continuous than that in the SG/BN composites. These morphological differences are consistent with a possible influence of the PDA-associated surface layer on the wettability and aggregation behavior of BN. The more compact and continuous morphology observed for the SG/P-BN composites may favor interfacial heat transfer and reduce defect-related electrical weak regions.

### 2.2. Thermal Properties Characterization

Efficient heat dissipation is essential for ensuring the long-term reliability of high-voltage power devices. During operation, switching losses, conduction losses, and localized electric field concentration continuously generate heat, which tends to accumulate near chips and insulating interfaces when the encapsulation material has limited thermal conductivity. Such localized heat accumulation increases temperature gradients and may accelerate thermal aging, interfacial delamination, carrier transport, and insulation degradation [37,38]. Therefore, improving the thermal stability, dimensional stability, and heat-transfer capability of silicone gel encapsulants is critical for high-voltage power module packaging. Based on the morphological differences observed above, the thermal degradation behavior, thermal expansion response, and heat-transfer performance of the P-BN-containing SG composites were further evaluated.

**Figure 3:**
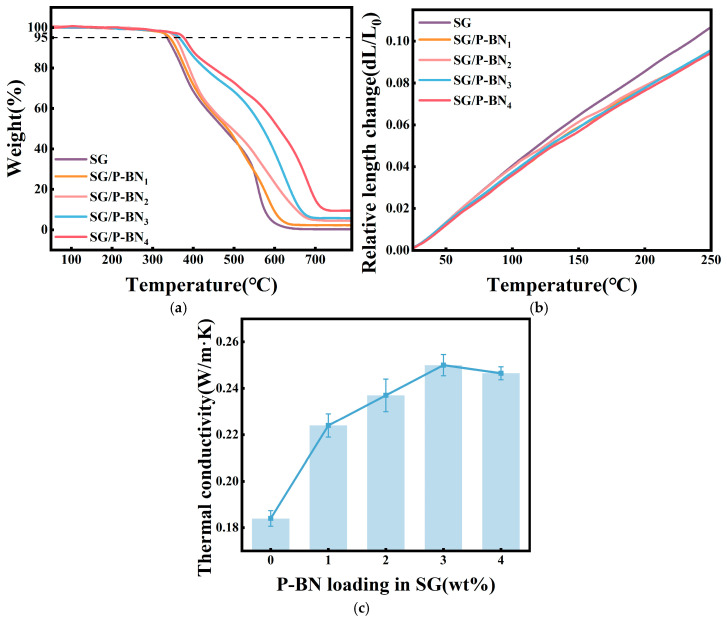
Thermal properties of pristine SG and SG/P-BN composites. (**a**) TGA curves of pristine SG and SG/P-BN composites. (**b**) Relative length change in pristine SG and SG/P-BN composites as a function of temperature. (**c**) Thermal conductivity of SG/P-BN composites with different P-BN contents. Error bars in (**c**) represent one standard deviation (*n* = 3).

The thermal stability of pristine SG and SG/P-BN composites was tested by TGA, and the results are presented in Figure 3a. All samples exhibit good thermal stability in the low-temperature region, with no obvious mass loss before approximately 300 °C, indicating that the incorporation of P-BN does not compromise the basic thermal stability of the silicone gel matrix. The characteristic thermal decomposition parameters derived from the TGA and DTG curves are summarized in Table 1. Pristine SG exhibits T_5%_ and T_10%_ values of 335.5 and 349.0 °C, respectively, with a maximum degradation rate temperature (T_max_) of 376.3 and 558.3 °C. After P-BN incorporation, the T_5%_ and T_10%_ values generally shift to higher temperatures. SG/P-BN_4_ exhibits the highest T_5%_ of 376.8 °C, compared with 335.5 °C for pristine SG. Meanwhile, the residual mass at 800 °C increases from 0.23% for pristine SG to 2.08–9.23% for the SG/P-BN composites. These quantitative results indicate that P-BN-containing SG composites exhibit improved thermal resistance under the investigated TGA conditions. This enhancement may be associated with the intrinsic thermal stability of the BN-based filler and the barrier effect of its layered structure, which can restrict polymer-chain motion and hinder the diffusion of volatile decomposition products. Moreover, the more uniform dispersion of P-BN within the SG matrix may enable the nanosheets to form a more effective physical barrier, thereby further constraining silicone-chain motion and the transport of volatile degradation products [39]. Therefore, the P-BN-containing composites exhibit enhanced thermal resistance under the investigated TGA conditions.

The coefficient of thermal expansion (CTE) is a key parameter for evaluating the dimensional stability of encapsulation insulating materials in power modules. A lower CTE can reduce thermal expansion mismatch among silicone gel, chips, ceramic substrates, and metal electrodes, thereby alleviating thermomechanical stress concentration at interfaces during thermal cycling and decreasing the risk of package cracking, interfacial delamination, and local electric field distortion [40]. The thermal expansion behavior of pristine SG and SG/P-BN composites was tested by TMA, and the results are presented in Figure 3b. The relative length change in all samples increases approximately linearly with temperature, indicating stable thermal expansion behavior within the tested temperature range. As summarized in Table 2, pristine SG exhibits a CTE of 468.9 μm/m·°C, whereas the CTE values of the SG/P-BN composites decrease to approximately 415 μm/m·°C after P-BN incorporation. This reduction suggests that the dispersed BN-based nanosheets may restrict the thermal motion and volumetric expansion of silicone molecular chains during heating. Notably, the CTE does not decrease monotonically with increasing P-BN content but tends to reach a plateau at relatively low filler loadings. This behavior may be associated with the dispersion state of P-BN, nanosheet orientation, and interfacial confinement effect within the silicone gel matrix. At moderate P-BN contents, the dispersed rigid nanosheets and associated interfacial regions may effectively restrict chain-segment mobility. However, further increasing the filler content may promote local filler–filler interactions or slight agglomeration, which weakens the efficiency of interfacial confinement and causes the CTE improvement to level off.

The heat-transfer performance of pristine SG and SG/P-BN composites was further tested by thermal conductivity measurement. As presented in Figure 3c, pristine SG exhibits a low thermal conductivity of approximately 0.183 W/m·K, while the incorporation of P-BN significantly improves the thermal conductivity of the composites. With increasing P-BN content, the thermal conductivity first increases and then slightly decreases. Specifically, the thermal conductivities of SG/P-BN_1_, SG/P-BN_2_, and SG/P-BN_3_ increase to approximately 0.224, 0.237, and 0.250 W/m·K, respectively, with SG/P-BN_3_ showing the highest value. When the P-BN content is further increased to SG/P-BN_4_, the thermal conductivity slightly decreases to approximately 0.246 W/m·K, but remains markedly higher than that of pristine SG. The increase in thermal conductivity may be related to the high intrinsic thermal conductivity of the BN-based nanosheets, which could facilitate the formation of heat-transfer pathways in the silicone gel matrix. In addition, the improved filler dispersion and more continuous filler–matrix morphology observed for the SG/P-BN composites may promote interfacial heat transfer and reduce the interruption of heat-conduction pathways caused by microstructural defects. The slight decrease at 4 wt% may be associated with local nanosheet stacking or agglomeration, which increases phonon scattering.

### 2.3. Electrical Properties Characterization

To verify the role of P-BN in modulating charge transport behavior, the trap energy level *E_T_* and trap density N_t_(E) were derived from the voltage decay curves obtained from surface potential decay (SPD) measurements using Equations (1) and (2) [41]:

(1) $$N_{t}\left(E\right)=\frac{4\varepsilon_{0}\varepsilon_{r}}{ek_{B}TL^{2}}\left|t\frac{dV_{s}(t)}{dt}\right|$$

(2) $$E_{T}=k_{B}Tln(v_{ATE}t)$$

Here, *e* is the charge of an electron, *L* is the thickness of the sample, *T* is the absolute temperature, *K_B_* is the Boltzmann constant, *V_s_*(*t*) is the potential at the sample surface at time *t*, *dV_s_*(*t*)/*dt* is the decay rate of the surface potential at time *t*, and *V_ATE_* is the charge escape frequency. In the present calculation, *T* = 298.15 K and *V_ATE_* = 1 × 10^11^ s^−1^ was used. All time values were expressed in seconds. The relative permittivity values used for SG, SG/P-BN_1_, SG/P-BN_2_, SG/P-BN_3_, and SG/P-BN_4_ were 2.73, 3.48, 3.52, 3.27, and 3.26, respectively, as measured at 25 °C and 1 kHz.

The measured surface-potential decay curves were fitted using a biexponential function:

(3) $$V_{S}\left(t\right)=A_{1}\exp\left(B_{1}t\right)+A_{2}exp(B_{2}t)$$

where *A*_1_, *A*_2_, *B*_1_, and *B*_2_ are fitting parameters. No additional smoothing was applied to the raw SPD data. The derivative *dV_s_* (*t*)/*dt* was analytically obtained from the fitted biexponential function and subsequently used to calculate the trap-density distribution.

The experimentally recorded time array ranged from 0 to 2400 s. The point at *t* = 0 was retained for biexponential fitting but excluded from the logarithmic energy transformation. Because the second higher-energy trap peak was not completely resolved within the 2400 s acquisition window, the fitted biexponential function was subsequently evaluated over an extended time array from 10^−1^ to 10^6^ s to estimate the higher-energy portion of the trap distribution. Therefore, the trap distribution corresponding to *t* > 2400 s represents a biexponential-model extrapolation rather than a directly experimentally resolved distribution.

The fitting-related uncertainty of the model-extrapolated deep-trap peak parameters was evaluated using residual bootstrap resampling with 1000 realizations. The 2.5th and 97.5th percentiles of the bootstrap distributions were used to determine the 95% confidence intervals. The peak parameters and their 95% bootstrap confidence intervals are summarized in Table 3. These confidence intervals quantify the fitting-related uncertainty conditional on the adopted biexponential model.

**Figure 4:**
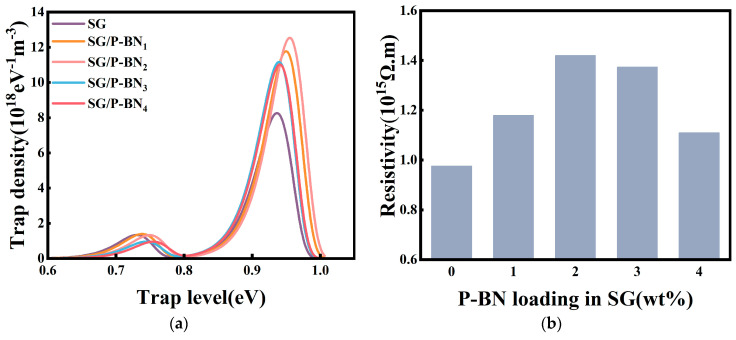
(**a**) Biexponential-model-derived trap level distributions of pristine SG and SG/P-BN composites with different P-BN loadings calculated from the measured surface potential decay curves. (**b**) Volume resistivity of silicone gel composites with different P-BN loadings.

The trap level distribution of pristine SG and SG/P-BN composites was derived from the measured surface potential decay (SPD) curves through biexponential fitting and time-scale extrapolation, and the results are presented in Figure 4a. All samples exhibit a distinct bimodal distribution, corresponding to low-energy shallow traps and high-energy deep traps. The shallow traps are mainly distributed in the range of approximately 0.70–0.78 eV, whereas the deep traps are concentrated at approximately 0.92–0.98 eV. It should be noted that the higher-energy deep trap peaks were obtained from the model-extrapolated portion of the fitted biexponential functions rather than being directly resolved within the 2400 s experimental acquisition window. Compared with pristine SG, the model-extrapolated results suggest that the incorporation of P-BN increases the deep trap density of the composites, with the maximum value reaching 1.25 × 10^19^ eV^−1^m^−3^. This behavior may be associated with the heterogeneous interfacial regions formed between P-BN and the silicone gel matrix, where local structural and chemical differences could modify the energy landscape for charge transport. Polar groups associated with the coated surface may also contribute to the formation of charge-trapping states.

The trap distribution also shows a clear dependence on P-BN content. Among the composites, SG/P-BN_2_ exhibits a relatively higher trap density in the deep-trap region, suggesting that an appropriate P-BN loading may construct a more uniform and effective interfacial trap structure. This may be beneficial for enhancing charge-trapping capability and inhibiting carrier migration. However, when the P-BN content is further increased, the deep trap density decreases, which may be associated with local filler agglomeration and interfacial inhomogeneity. Excessive filler loading can introduce additional structural defects and reduce the effectiveness of interfacial traps; in some regions, these interfacial sites may even evolve from charge-trapping centers into potential charge transport pathways, thereby weakening their confinement effect on carriers. Overall, the model-extrapolated SPD results suggest that P-BN may regulate the trap level distribution of silicone gel by increasing the density of deep traps, which may contribute to suppressing space charge accumulation and carrier transport.

Volume resistivity was further measured to test the ability of the SG/P-BN composites to suppress leakage current and charge migration. As a key macroscopic parameter for dielectric insulation materials, higher volume resistivity generally indicates more effective inhibition of carrier transport under an applied electric field. In this study, the volume resistivity of pristine SG and SG/P-BN composites with different P-BN contents was calculated from the leakage current measured using a three-electrode configuration according to Equation (4) [42]:

(4) $$\rho =\frac{US}{IL}$$

Here, *U* is the applied voltage, *S* is the sample area, *I* is the leakage current, and *L* is the sample thickness.

Figure 4b shows the volume resistivity of pristine SG and SG/P-BN composites with different P-BN contents. The volume resistivity first increases and then decreases with increasing P-BN loading. Pristine SG exhibits a volume resistivity of 9.76 × 10^14^ Ω·m, whereas the incorporation of P-BN significantly improves the resistivity of the composites. When the P-BN content reaches 2 wt%, SG/P-BN_2_ shows the highest volume resistivity of 1.42 × 10^15^ Ω·m, corresponding to an increase of approximately 45.4% compared with pristine SG. This improvement indicates that an appropriate amount of P-BN can effectively suppress carrier migration and enhance the electrical insulation performance of silicone gel.

However, when the content of P-BN is further increased to 3 and 4 wt%, the volume resistivity decreases, although it remains higher than that of pristine SG. This decrease may be related to the reduced filler spacing, local filler agglomeration, and increased interfacial defects at higher filler loadings. Under these conditions, excessive P-BN may partially weaken the charge-trapping effect and promote the formation of localized charge transport pathways, thereby reducing the resistivity enhancement. Overall, the results demonstrate that moderate P-BN loading is beneficial for improving the volume insulation performance of SG, with SG/P-BN_2_ exhibiting the optimal resistivity. This trend is consistent with the model-extrapolated trap-distribution analysis and suggests a possible association between the interfacial charge-trapping environment and the observed improvements in volume resistivity.

**Figure 5:**
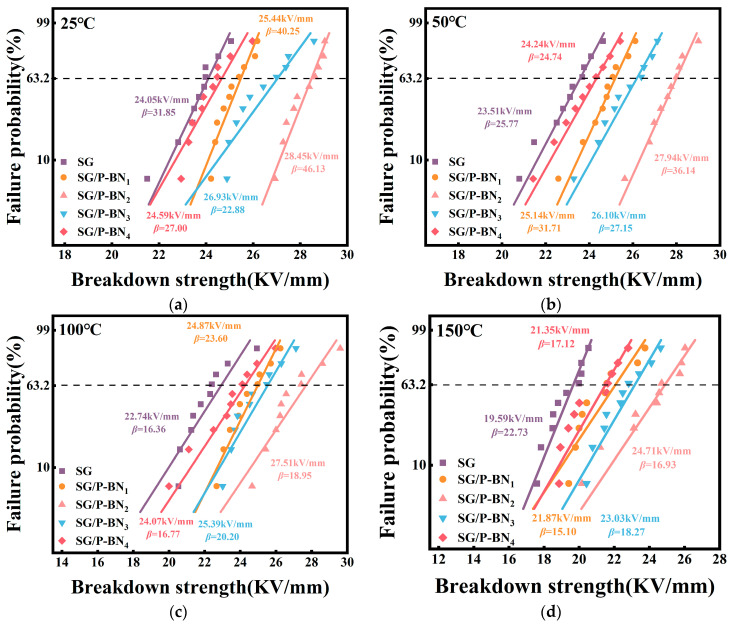
Weibull distributions of breakdown strength for pristine SG and SG/P-BN composites measured at (**a**) 25 °C, (**b**) 50 °C, (**c**) 100 °C, and (**d**) 150 °C.

The breakdown strength of pristine SG and SG/P-BN composites was further tested at 25, 50, 100, and 150 °C using Weibull statistical analysis, as shown in Figure 5. The characteristic breakdown strength at a failure probability of 63.2% was used as the evaluation parameter. The incorporation of P-BN significantly improves the breakdown strength of silicone gel over the entire tested temperature range, and the enhancement shows a clear dependence on P-BN content. Among all samples, SG/P-BN_2_ exhibits the highest characteristic breakdown strength among the investigated compositions, with characteristic breakdown strengths of 28.45, 27.94, 27.51, and 24.71 kV/mm at 25, 50, 100, and 150 °C, respectively. These values are higher than those of pristine SG, which are 24.05, 23.51, 22.74, and 19.59 kV/mm at the corresponding temperatures. This result indicates that an appropriate amount of P-BN not only enhances the room-temperature breakdown resistance of silicone gel but also maintains a higher characteristic breakdown strength at elevated temperatures.

As the temperature increases, the breakdown strength of all samples gradually decreases, accompanied by an overall reduction in the Weibull shape parameter β. This suggests that high temperature weakens the intrinsic breakdown resistance of the materials and increases the dispersion and uncertainty of breakdown failure. The decrease in breakdown strength can be attributed to enhanced molecular chain mobility, enlarged free volume, and intensified thermal excitation of carriers in the silicone gel matrix at elevated temperatures. These factors facilitate charge injection, migration, and accumulation, thereby promoting the initiation and propagation of localized interfacial charge transport channels. In addition, thermal effects may amplify microscopic inhomogeneities at the filler–matrix interface, resulting in a more complex local electric field distribution and making breakdown paths more sensitive to microdefects, interfacial structures, and local charge accumulation. Therefore, the overall decrease in β with increasing temperature indicates increased statistical scatter in the breakdown data at elevated temperatures. This change in data dispersion should be distinguished from the temperature dependence of the characteristic breakdown strength η.

At 150 °C, SG/P-BN_2_ maintains a characteristic breakdown strength of 24.71 kV/mm, which is higher than the 19.59 kV/mm of pristine SG. However, the β value of SG/P-BN_2_ is lower than that of pristine SG, indicating greater scatter in its breakdown data. Therefore, the higher η value is interpreted as an increase in characteristic breakdown strength rather than as direct evidence of improved statistical breakdown reliability.

Notably, the breakdown strength of SG/P-BN_2_ decreases by only approximately 13.1% from 25 to 150 °C, which is lower than the decrease in pristine SG over the same temperature range. This indicates that the P-BN-containing composite exhibits reduced thermal-field-induced deterioration in characteristic breakdown strength under the investigated conditions. The higher breakdown strength of SG/P-BN_2_ may be associated with the more uniform filler dispersion and more continuous filler–matrix morphology observed by SEM, which could reduce defect-related local electric-field distortion. Meanwhile, the model-extrapolated trap results suggest that additional deep charge-trapping states may be introduced after P-BN incorporation; these states could capture injected electrons and restrict carrier migration, thereby potentially delaying the formation of breakdown channels. However, when the P-BN content is further increased, local agglomeration and interfacial inhomogeneity may reappear, acting as weak regions for electric field concentration and defect-induced breakdown [20]. Overall, SG/P-BN_2_ achieves an optimal balance among filler dispersion, interfacial regulation, and possible charge transport suppression, resulting in the best breakdown performance over a wide temperature range.

Dielectric stability over a wide temperature and frequency range is critical for electric-field regulation, dielectric loss control, and long-term insulation reliability [43]. In practical packaging structures, local electric field distortion at triple-junction interfaces is closely related to the dielectric mismatch among the ceramic substrate, metal electrode, and silicone gel encapsulant. As described by Equation (5), increasing the dielectric constant of silicone gel can reduce the dielectric constant difference between adjacent insulating media, thereby helping to alleviate local electric field concentration at the triple-junction region [44]:

(5) $$E=\frac{U}{d_{1}+d_{2}\frac{\varepsilon_{1}}{\varepsilon_{2}}}$$

Here, *U* represents the applied voltage; *d*_1_ and *d*_2_ are the thicknesses of the organic silicone gel and the ceramic substrate, respectively. *ε*_1_ and *ε*_2_ are the dielectric constants of the silicone gel and the ceramic substrate, respectively. It can thus be concluded that increasing the dielectric constant of the silicone gel is effective in mitigating localized electric field concentration at the triple junctions of high-voltage devices.

**Figure 6:**
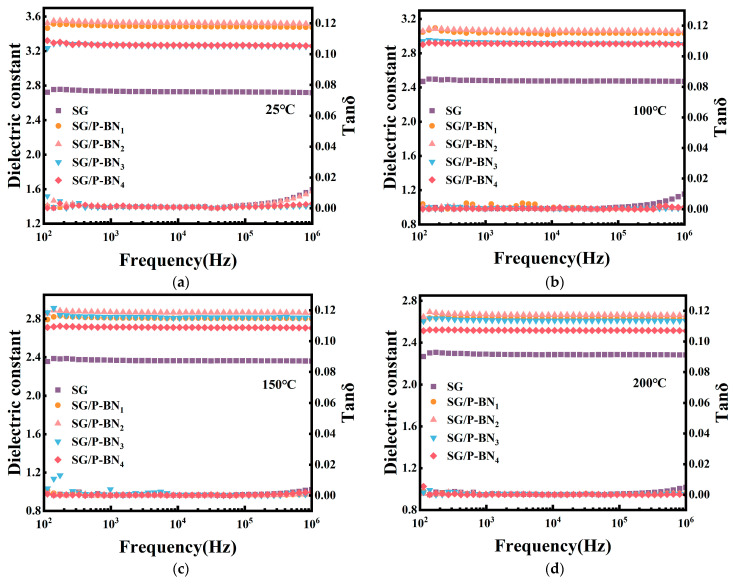
Dielectric properties of SG and SG/P-BN composites at different temperatures. Frequency-dependent dielectric constant and dielectric loss tangent of SG and SG/P-BN composites measured from 10^2^ to 10^6^ Hz at (**a**) 25 °C, (**b**) 100 °C, (**c**) 150 °C, and (**d**) 200 °C.

Therefore, the frequency-dependent dielectric constant and dielectric loss of pristine SG and SG/P-BN composites were systematically investigated from 25 to 200 °C, as shown in Figure 6. In the frequency range of 10^2^–10^6^ Hz, all samples exhibit weak frequency dependence in dielectric constant, indicating a stable polarization response without obvious low-frequency electrode polarization or high-frequency relaxation instability. Compared with pristine SG, the incorporation of PDA-modified BN increases the dielectric constant of the composites at all tested temperatures. For example, at room temperature, the dielectric constant of pristine SG is approximately 2.73, whereas that of the SG/P-BN composites reaches up to 3.52. Although the dielectric constant slightly decreases as the temperature increases to 100, 150, and 200 °C, the SG/P-BN composites still maintain higher values than pristine SG, demonstrating improved polarization capability and good dielectric stability at elevated temperatures.

The enhanced dielectric constant may be associated with the combined contributions of the BN-based nanosheets and interfacial polarization. The incorporation of BN-based fillers may increase the effective dielectric response of the composite system, while the more continuous filler–matrix morphology observed in the P-BN-containing composites may favor the formation of interfacial polarization regions. This moderate increase in dielectric constant is beneficial for reducing dielectric mismatch among the ceramic substrate, metal electrode, and silicone gel encapsulant, thus helping to alleviate local electric field concentration near triple-junction interfaces. However, the dielectric constant does not increase continuously with increasing P-BN content. When the filler loading is too high, local stacking of BN nanosheets or filler-rich regions may reduce the effective interfacial area and introduce microstructural inhomogeneity, limiting further improvement in dielectric response.

The dielectric loss results show that all samples maintain low tanδ values over the frequency range of 10^2^–10^6^ Hz, indicating that P-BN does not introduce significant conductive loss or polarization relaxation loss into the silicone gel matrix. Pristine SG shows a slight increase in tanδ in the high-frequency region, especially at room temperature and 100 °C, which may be associated with more pronounced segmental dipole response or interfacial dissipation under high-frequency electric fields. In contrast, the SG/P-BN composites exhibit more stable dielectric loss behavior. Even when the temperature increases to 200 °C, the composites maintain low tanδ values and stable frequency response, suggesting good high-temperature dielectric reliability. This stability may be associated with the confinement effect of the dispersed BN-based nanosheets and the interfacial structure of the P-BN-containing composites, which could restrict the thermal motion of silicone gel molecular chains and hinder the formation of continuous carrier-migration pathways. Overall, the P-BN-containing composites exhibit increased dielectric constant while maintaining low dielectric loss, indicating favorable dielectric properties under the investigated temperature and frequency ranges.

Although macroscopic insulation parameters, such as breakdown strength and volume resistivity, reflect the overall dielectric strength and charge-blocking capability of silicone gel, they cannot fully represent its insulation reliability in practical high-voltage power module packaging. In actual device structures, triple-junction interfaces are inevitably formed among the metal electrode, ceramic substrate, and silicone gel encapsulant. Owing to dielectric mismatch, geometric edge effects, and interfacial microdefects, these regions are prone to local electric field enhancement and thus become preferential sites for partial discharge. Unlike bulk breakdown under a relatively uniform electric field, partial discharge is usually localized and cumulative. The generated high-energy electrons, local thermal impact, and interfacial erosion can gradually damage the insulation integrity of the silicone gel/electrode/ceramic interface, eventually leading to insulation degradation. Therefore, in addition to conventional breakdown and resistivity measurements, evaluating the partial discharge behavior of silicone gel under a controlled triple-junction configuration provides complementary information on its comparative interfacial discharge characteristics.

**Figure 7:**
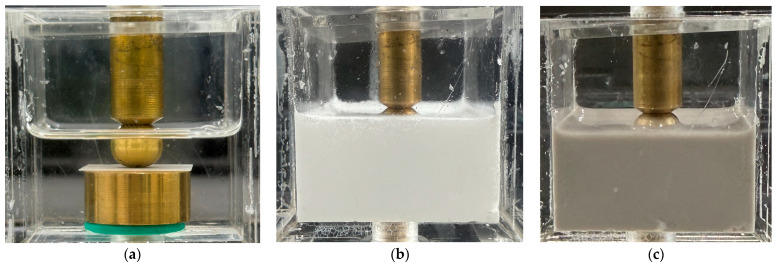
Photographs of the simplified triple-junction laboratory configuration for partial discharge testing. (**a**) pristine silicone gel, (**b**) silicone gel composite containing 2 wt% unmodified BN, and (**c**) silicone gel composite containing 2 wt% P-BN.

Based on the above considerations, a simplified triple-junction laboratory configuration was constructed by inserting an AlN ceramic sheet into a sphere–plate electrode arrangement to investigate partial discharge behavior under a controlled electrode/ceramic/silicone gel interfacial geometry. According to the macroscopic insulation results discussed above, SG/P-BN_2_, which exhibited the best overall performance, was selected as the representative modified composite. Pristine SG and SG/BN_2_ containing 2 wt% pristine BN were used as control samples. The encapsulated samples are shown in Figure 7. The partial discharge inception voltage (PDIV) of the three samples was first measured under this triple-junction configuration. To further test the discharge stability and phase-resolved discharge behavior under overvoltage conditions, 1000 partial discharge pulses were recorded at 1.5 times the PDIV of each sample. The background noise was approximately 100 mV; therefore, based on a signal-to-noise ratio of 3:1, signals with amplitudes higher than 300 mV were identified as partial discharge signals.

**Figure 8:**
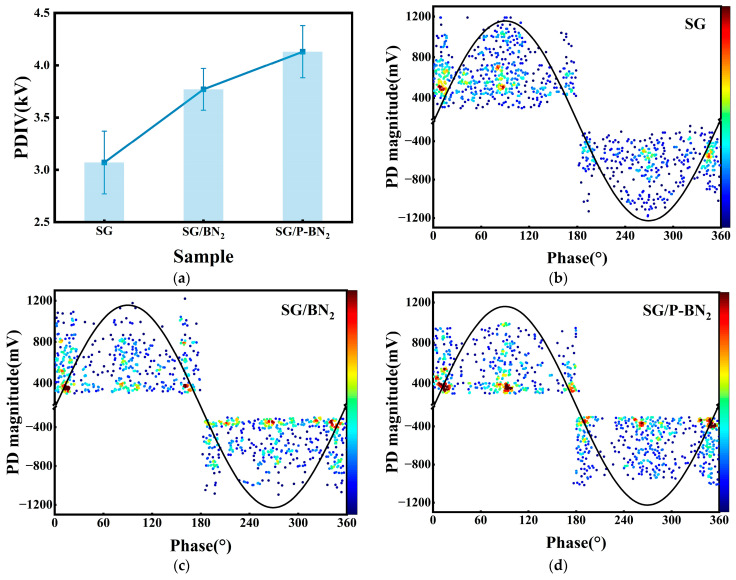
Partial discharge characteristics of silicone gel composites under a simplified triple-junction laboratory configuration. (**a**) Partial discharge inception voltage (PDIV) of SG, SG/BN_2_, and SG/P-BN_2_. (**b**–**d**) Phase-resolved partial discharge patterns of (**b**) pristine SG, (**c**) SG/BN_2_, and (**d**) SG/P-BN_2_ recorded over 1000 discharge events. Error bars in (**a**) represent one standard deviation (*n* = 5).

As shown in Figure 8a, the PDIV of pristine SG is approximately 3.1 kV, indicating that local electric field distortion at the electrode/ceramic/silicone gel triple-junction interface can preferentially trigger discharge initiation. After the introduction of 2 wt% pristine BN, the PDIV of SG/BN_2_ increases to approximately 3.8 kV, showing that SG/BN_2_ exhibits a higher PDIV than pristine SG under the investigated configuration. Notably, SG/P-BN_2_ exhibits the highest PDIV of approximately 4.1 kV, corresponding to an increase of about 32% compared with pristine SG. The higher PDIV of SG/P-BN_2_ than that of SG/BN_2_ at the same nominal filler loading shows that the surface-treated composite exhibits improved partial-discharge inception characteristics under the investigated configuration. This difference is consistent with the more uniform filler distribution and more continuous morphology observed for the SG/P-BN composites.

The phase-resolved partial discharge patterns in Figure 8b–d further reveal the discharge development characteristics of different samples under overvoltage conditions. For all samples, the discharge pulses are not uniformly distributed over the entire phase range but are mainly concentrated near the voltage polarity transition region and the voltage peak region. Near the polarity transition region, the applied electric field changes direction rapidly, and residual interfacial charges generated in the previous half-cycle can be superimposed with the reverse electric field, promoting discharge re-initiation at the triple junction. Near the voltage peak region, the instantaneous electric field strength is relatively high and can more easily exceed the partial discharge threshold at interfacial defects or micro voids. Therefore, the concentrated discharge distribution in these characteristic phase regions suggests that residual charge accumulation, rapid electric field variation, and local defects at the triple-junction interface are the main factors governing partial discharge behavior.

For pristine SG, the discharge pulses are broadly distributed within the characteristic phase regions, accompanied by numerous discrete high-amplitude discharge events with amplitudes approaching ±1200 mV. This indicates that pristine SG has limited ability to suppress residual charge accumulation and discharge channel development at the triple-junction interface, resulting in relatively random and unstable discharge behavior. After the introduction of pristine BN, the number of high-amplitude discharge pulses in SG/BN_2_ decreases, and the discharge events are mainly distributed in the medium-amplitude range, indicating that BN can partially reduce the intensity of partial discharge. However, scattered discharge points and localized high-density regions are still observed, which may be related to the limited interfacial compatibility between pristine BN and the silicone gel matrix. Filler agglomeration, interfacial voids, or dielectric discontinuities may still act as weak regions for local electric field concentration.

In contrast, SG/P-BN_2_ exhibits more stable phase-resolved discharge behavior. Its discharge pulses are mainly concentrated in the low-to-medium amplitude range, and high-amplitude discrete pulses are significantly reduced. Meanwhile, the discharge-dense regions show a narrower amplitude window, indicating that the development of partial discharge is effectively constrained. The improved PRPD characteristics of SG/P-BN_2_ may be associated with the combined effects of its more uniform filler distribution, more continuous filler matrix morphology, and possible changes in charge-transport behavior. These factors could reduce defect-related local electric-field enhancement and residual-charge accumulation during polarity reversal. However, the specific PDA-related mechanisms cannot be conclusively established from the present control experiments. Therefore, SG/P-BN_2_ exhibits the highest PDIV and the most stable PRPD characteristics among the samples investigated, demonstrating improved partial-discharge behavior under the tested triple-junction laboratory configuration.

The present study focuses on laboratory-scale thermal and electrical characterization of P-BN-containing silicone gel composites. The increase in thermal conductivity represents a measurable material-level improvement but does not directly establish reduced device temperature or enhanced thermal management in a practical power module. Similarly, the simplified triple-junction configuration provides comparative information on interfacial partial discharge behavior but does not reproduce the complete electrical, thermal, mechanical, and geometric conditions of an operating device. A further limitation is the absence of a complete SG/BN control series for the thermal, surface potential decay, volume resistivity, dielectric, and breakdown measurements. Although matched SG/BN and SG/P-BN comparisons were included in the SEM observations over the investigated filler range and in the partial-discharge tests at 2 wt%, these controls are insufficient to quantitatively separate the contributions of BN itself, the PDA-associated surface layer, filler dispersion, interfacial morphology, and possible changes in the curing process. Therefore, the PDA-specific mechanisms discussed in this work should be regarded as possible interpretations rather than conclusively demonstrated effects, whereas the measured thermal and electrical improvements should be interpreted as properties of the P-BN-containing SG composites. In addition, mechanical properties, rheological and processing behavior, curing state, interfacial adhesion, thermal cycling, humidity resistance, and long-term electrothermal aging were not evaluated in the present study. These aspects, together with matched SG/BN and SG/P-BN control series, require further investigation before the individual contribution of the PDA-associated coating and the practical encapsulation performance and long-term reliability of the composites can be conclusively established.

## 3. Conclusions

In this study, polydopamine-modified boron nitride (P-BN) was introduced into silicone gel to investigate the material-level thermal and electrical properties of the composites. SEM comparisons showed that the P-BN-containing composites exhibited fewer filler agglomerates and a more continuous filler–matrix morphology than the corresponding SG/BN composites, while the intrinsic crystalline structure of h-BN was preserved after surface treatment. The SG/P-BN composites exhibited enhanced thermal stability, reduced coefficient of thermal expansion, and improved heat-transfer capability. The SG/P-BN composites exhibited increased thermal conductivity, with a maximum value of approximately 0.250 W/m·K for SG/P-BN_3_, representing an increase of approximately 36.6% compared with pristine SG. In addition, the model-extrapolated SPD analysis suggested that P-BN-containing composites exhibit composition-dependent changes in the higher energy trap distribution, which may influence charge transport in the composites. The SG/P-BN_2_ sample exhibited characteristic breakdown strengths of 28.45, 27.94, 27.51, and 24.71 kV/mm at 25, 50, 100, and 150 °C, respectively, maintaining higher characteristic breakdown strength than pristine SG over the investigated temperature range. In addition, a simplified triple-junction laboratory configuration was used to comparatively evaluate the partial discharge behavior of the composites. Under this configuration, the partial discharge inception voltage of SG/P-BN_2_ increased from approximately 3.1 kV for pristine SG to 4.1 kV, accompanied by a clear reduction in high-amplitude discharge events. These improvements may be associated with the combined effects of BN incorporation, filler dispersion, interfacial morphology, heat-transfer pathways, and changes in charge-transport behavior. However, because a complete SG/BN control series was not available, the individual contribution of the PDA-associated surface layer cannot be conclusively established. Therefore, the present results demonstrate increased material-level thermal conductivity and characteristic breakdown strength of the P-BN-containing silicone gel composites, together with higher PDIV and more stable PRPD behavior under the investigated triple-junction laboratory configuration. Future work should further evaluate the mechanical and processing properties, curing behavior, interfacial adhesion, and long-term stability of the composites under thermal aging, electro-thermal cycling, humidity exposure, and practical power module packaging conditions.

## 4. Materials and Methods

### 4.1. Reagents

The two-component addition-curing silicone gel (5298) was purchased from Hubei Huitian New Materials Co., Ltd. (Xiangyang, China). Component A contained polymethyl vinyl silicone oil as the base silicone oil, while Component B contained side-chain hydrogen-containing silicone oil as the crosslinking agent. Hexagonal boron nitride (h-BN, 1–2 μm, ≥99.9%) and dopamine hydrochloride (≥98%) were obtained from Shanghai Aladdin Biochemical Technology Co., Ltd. (Shanghai, China). Tris (hydroxymethyl)aminomethane (Tris, 99.9%, biotechnology grade) and anhydrous ethanol were supplied by Shanghai Macklin Biochemical Technology Co., Ltd. (Shanghai, China). Deionized water was prepared in the laboratory. All reagents were used as received without further purification.

### 4.2. Preparation of SG/P-BN Composites

#### 4.2.1. Preparation of Polydopamine-Modified h-BN

Polydopamine-modified hexagonal boron nitride (P-BN) was prepared through the oxidative self-polymerization of dopamine under weakly alkaline conditions. First, 230 mg of Tris was dissolved in 185 mL of deionized water under continuous stirring to obtain a Tris buffer solution with a pH of approximately 8.5. Then, 65 mL of anhydrous ethanol was added, and the mixture was stirred until a homogeneous water/ethanol mixed solvent was formed. Subsequently, 10 g of pristine h-BN was added to the mixed solvent, preliminarily dispersed with a glass rod, and ultrasonicated at room temperature for 30 min to promote uniform dispersion. Dopamine hydrochloride (500 mg) was then added to the h-BN suspension, and the mixture was magnetically stirred at room temperature for 10 h. During this process, dopamine underwent oxidative self-polymerization in the weakly alkaline medium, leading to the formation of a PDA coating on the h-BN surface. After the reaction, the suspension was centrifuged at 9000 rpm for 10 min, and the precipitate was collected after removing the supernatant. The precipitate was washed with deionized water and centrifuged three times to remove residual dopamine, Tris, and other soluble impurities. Finally, the purified product was dried in a vacuum oven at 75 °C for 12 h, ground using a ceramic mortar, and stored in a desiccator for further use.

#### 4.2.2. Fabrication of SG/P-BN Composites

**Figure 9:**
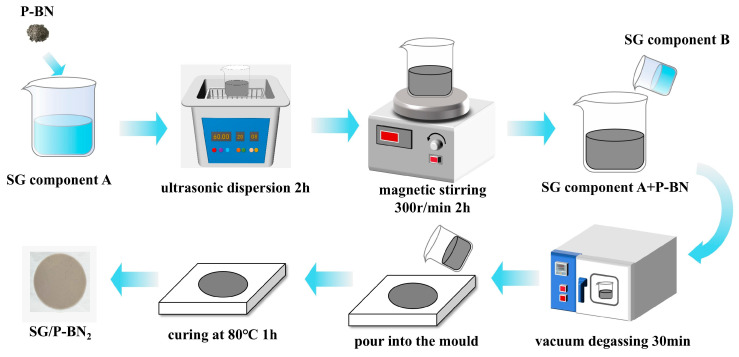
Schematic illustration of the fabrication process of SG/P-BN composite insulating materials.

A specified amount of silicone gel Component A was first added to a beaker, followed by the addition of P-BN according to the designed mass fraction. The mixture was ultrasonically dispersed for 2 h to promote the initial dispersion of P-BN in Component A. After ultrasonication, the mixture was magnetically stirred at 300 rpm for 2 h at room temperature to further improve the dispersion uniformity of P-BN. Subsequently, silicone gel Component B was added at a mass ratio of 1:1 relative to Component A, and the mixture was stirred for another 15 min to ensure complete mixing of the two components. The obtained mixture was then degassed under vacuum for 30 min to remove bubbles introduced during ultrasonication and stirring. Finally, the degassed mixture was poured into molds and cured at 80 °C for 1 h to obtain the SG/P-BN composite insulating materials. Using this procedure, composites with P-BN contents of 0, 1, 2, 3, and 4 wt% were prepared and denoted as SG, SG/P-BN_1_, SG/P-BN_2_, SG/P-BN_3_, and SG/P-BN_4_, respectively. The preparation process is schematically illustrated in Figure 9.

### 4.3. Characterization of the Silicone Gel Composites

#### 4.3.1. Structural and Morphological Characterization of P-BN and SG/P-BN Composites

Fourier transform infrared spectroscopy (FTIR, Nicolet iS20, Thermo Scientific, Waltham, MA, USA) was used to characterize the chemical structures of h-BN and P-BN. The spectra were recorded at room temperature over a wavenumber range of 400–4000 cm^−1^. X-ray diffraction (XRD, SmartLab, 9 kW, Rigaku Corporation, Tokyo, Japan) was performed to analyze the crystalline structures of h-BN and P-BN in the 2θ range of 5–70°. Thermogravimetric analysis (TGA, STA 449 F3, Netzsch, Selb, Germany) was conducted to test the thermal weight-loss behavior of h-BN and P-BN under a nitrogen atmosphere from room temperature to 800 °C at a heating rate of 10 °C/min. Transmission electron microscopy (TEM, JEM-F200, JEOL, Tokyo, Japan) was employed to observe the morphology and surface microstructure of the modified BN, with a representative scale bar of 5 nm. In addition, scanning electron microscopy (SEM, GAIA3 Ga, TESCAN, Brno, Czech Republic) was used to examine the fractured cross-sectional morphology of silicone gel composites filled with pristine BN and P-BN. Before SEM observation, the samples were cryo-fractured in liquid nitrogen to obtain fresh cross-sections and then sputter-coated with gold. The filler dispersion and filler–matrix interfacial morphology of the SG/BN and SG/P-BN composites were comparatively examined.

#### 4.3.2. Thermal Properties of SG/P-BN Composites

Thermogravimetric analysis (TGA, STA 449 F3, Netzsch, Selb, Germany) was performed to test the thermal weight-loss behavior of pristine SG and SG/P-BN composites with different P-BN contents. The measurements were conducted under a nitrogen atmosphere from room temperature to 800 °C at a heating rate of 10 °C/min. The thermal expansion behavior of the samples was measured using a thermomechanical analyzer (TMA Q400, TA Instruments, New Castle, DE, USA) over the temperature range of 25–250 °C at a heating rate of 10 °C/min. The coefficient of thermal expansion (CTE) was calculated from the linear fitting of the relative length change as a function of temperature. Three replicate measurements were performed for each composition. The thermal conductivity of the samples was measured at room temperature using a thermal constants analyzer (Hot Disk TPS 2500S, Hot Disk, Gothenburg, Sweden). Thermal conductivity measurements were performed three times for each composition.

#### 4.3.3. Electrical Insulation Properties of SG/P-BN Composites

**Figure 10:**
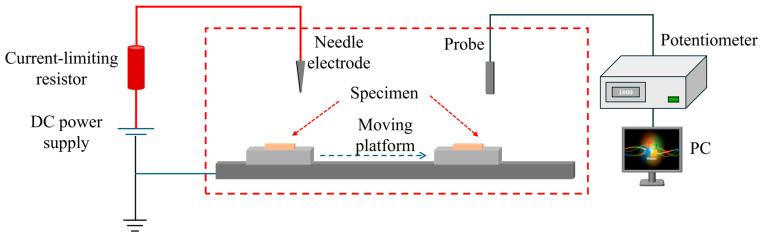
Schematic diagram of the surface potential decay (SPD) measurement system.

Surface potential decay (SPD) measurements were performed to investigate the microscopic charge transport behavior of pristine SG and SG/P-BN composites. The experimental setup is shown in Figure 10. Samples with a thickness of 1 mm were tested at 25 °C. During the test, the sample was first placed 5 mm below the corona charging needle electrode and charged at −10 kV for 15 min. After charging, the sample was rapidly transferred to a position 3 mm below the electrostatic probe, and the surface potential decay data were recorded for 40 min after the removal of the charging voltage. The measured SPD curves were fitted using a biexponential function without additional smoothing, and the analytical derivative of the fitted function was used to calculate the trap-density distribution. Because the higher-energy trap peak was not completely resolved within the experimental acquisition window, the fitted biexponential function was extrapolated over an extended time scale to estimate the higher-energy portion of the trap distribution. The results beyond the experimental acquisition window were therefore regarded as model-extrapolated estimates rather than directly experimentally resolved data. Residual bootstrap resampling was employed to evaluate the fitting-related uncertainty of the extrapolated peak parameters under the adopted biexponential model.

The volume resistivity of the samples was measured using a three-electrode configuration connected to a Keithley 6514 electrometer. During the test, a DC voltage of 10 kV was applied for 40 min, and the leakage current was recorded. Five measurements were performed for each sample, and the average value was used to calculate the volume resistivity. The breakdown strength of the samples was measured at room temperature, 50 °C, 100 °C, and 150 °C using a plate–plate electrode configuration. An AC voltage was applied at a rising rate of 1 kV/s until breakdown occurred. Considering the statistical dispersion of breakdown failure, ten valid breakdown data points were collected for each sample, and the breakdown strength was analyzed using a two-parameter Weibull distribution model. The dielectric properties of pristine SG and SG/P-BN composites with different P-BN contents were measured using a broadband dielectric spectrometer (Concept 80, Novocontrol, Montabaur, Germany). The relative permittivity and dielectric loss were recorded at room temperature, 100 °C, 150 °C, and 200 °C over the frequency range of 10^2^–10^6^ Hz.

**Figure 11:**
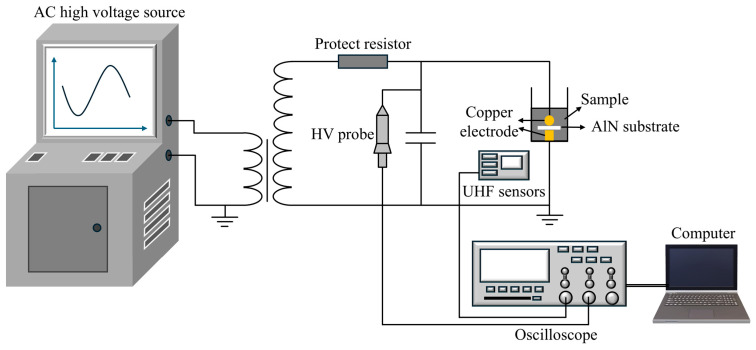
Schematic diagram of the partial discharge measurement system based on a sphere–ceramic substrate–plate electrode configuration.

A sphere–ceramic substrate–plate electrode partial discharge testing platform was constructed, as shown in Figure 11. During the test, silicone gel samples were encapsulated in a mold to form a controlled triple-junction geometry among the electrode, ceramic substrate, and silicone gel. An AlN ceramic sheet served as the ceramic insulating component in this simplified laboratory configuration. Partial discharge signals were detected using an ultra-high-frequency (UHF) sensor. The partial discharge inception voltage (PDIV) of composites with different compositions was measured. PDIV measurements were performed five times for each composition. In addition, to test the discharge behavior under overvoltage conditions, 1000 discharge signals were recorded for each sample at 1.5 times its corresponding PDIV, and the phase-resolved partial discharge characteristics were further analyzed.

## Figures and Tables

**Table 1:** TGA/DTG quantitative parameters of pristine SG and SG/P-BN composites.

Sample	T_5%_ (°C)	T_10%_ (°C)	DTG Peak1 (°C)	DTG Peak2 (°C)	Residual Mass at 800 °C (%)
SG	335.5	349.0	376.3	558.3	0.23
SG/P-BN_1_	342.1	357.6	375.3	582.5	2.08
SG/P-BN_2_	355.8	369.3	386.5	591.8	4.43
SG/P-BN_3_	364.9	383.7	378.3	622.8	5.62
SG/P-BN_4_	376.8	393.4	385.8	680.5	9.23

**Table 2:** The coefficient of thermal expansion (CTE) of pristine SG and SG/P-BN composites. Data are presented as mean ± standard deviation (SD) (*n* = 3).

Sample	CTE(μm/m·°C)
SG	468.9 ± 8.3
SG/P-BN_1_	420.3 ± 6.5
SG/P-BN_2_	414.6 ± 5.8
SG/P-BN_3_	420.4 ± 7.1
SG/P-BN_4_	414.8 ± 6.2

**Table 3:** Biexponential-model-extrapolated deep-trap peak parameters and their 95% confidence intervals obtained by residual bootstrap resampling.

Sample	Peak Energy (eV)	95% CI (eV)	Peak Density (10^18^ eV^−1^m^−3^)	95% CI (10^18^ eV^−1^m^−3^)
SG	0.9363	0.9325–0.9407	8.261	8.237–8.285
SG/P-BN_1_	0.9498	0.9474–0.9560	11.78	11.75–11.79
SG/P-BN_2_	0.9541	0.9420–0.9610	12.53	12.50–12.61
SG/P-BN_3_	0.9393	0.9338–0.9463	11.16	11.12–11.20
SG/P-BN_4_	0.9407	0.9343–0.9452	11.02	10.99–11.06

## Data Availability

The data presented in this study are available on request from the corresponding author.
